# Nutritional Management of Moderate- and Late-Preterm Infants Commenced on Intravenous Fluids Pending Mother's Own Milk: Cohort Analysis From the DIAMOND Trial

**DOI:** 10.3389/fped.2022.817331

**Published:** 2022-03-31

**Authors:** Tanith Alexander, Michael Meyer, Jane E. Harding, Jane M. Alsweiler, Yannan Jiang, Clare Wall, Mariana Muelbert, Frank H. Bloomfield, The DIAMOND Study Group

**Affiliations:** ^1^Liggins Institute, University of Auckland, Auckland, New Zealand; ^2^Neonatal Unit, Kidz First, Middlemore Hospital, Counties Mankau Health, Auckland, New Zealand; ^3^Newborn Services, Auckland City Hospital, Auckland, New Zealand; ^4^The Department of Paediatrics: Child and Youth Health, University of Auckland, Auckland, New Zealand; ^5^Department of Statistics, Faculty of Science, University of Auckland, Auckland, New Zealand; ^6^Department of Nutrition, Faculty of Medical and Health Sciences, University of Auckland, Auckland, New Zealand

**Keywords:** moderate preterm, late preterm, nutrition, breastmilk, intravenous fluid, Māori

## Abstract

**Background:**

Exclusive breastmilk is the desired enteral nutrition for babies born moderate- and late-preterm between 32^+0^ and 36^+6^ weeks' gestation; however, this goal is often difficult to achieve.

**Methods:**

A prospective cohort of babies 32^+0^ −35^+6^ weeks' gestation enrolled in the DIAMOND trial were randomized to a condition specifying that babies should receive mother's own milk (MOM) as the only enteral feed. Factors associated with the successful transition to MOM, defined as MOM being the sole enteral feeding at the time of the first cessation of intravenous (IV) fluids, were investigated by logistic regression. Time to commencement of a milk other than MOM was analyzed by Kaplan–Meier survival curves.

**Results:**

A total of 151 eligible babies (60% boys) were included, 93 (63%) of whom successfully transitioned from IV fluids onto MOM only. Alternative sources of milk, mostly formula, were used to transition from IV fluids onto enteral feeds more often in multiples and Māori, and was commenced earlier in Māori than other ethnicities (*p* = 0.007) and in late-preterm compared with moderate-preterm babies (p=0.01). Receiving exclusively breastmilk at discharge was more likely for babies who successfully transitioned from IV fluids onto MOM only [OR (95% confidence intervals) 4.9 (2.3–10.6)] and who received only MOM in the first week after birth [4.8 (2.2–10.4)], both *p* < 0.0001. Receiving breastmilk exclusively at discharge was less likely for Māori than Caucasian babies [0.2 (0.1–0.6), *p* < 0.0006]. There was no difference in the use of alternative sources of milk in babies who received parenteral nutrition or dextrose or between small-for-gestational-age and appropriate-for-gestational-age babies.

**Conclusions:**

Despite an intention to provide only MOM, significant numbers of moderate- and late-preterm babies received formula to transition from IV fluids, and this differed by ethnicity. The drivers underlying these decisions require further investigation. These data highlight an urgent need for quality initiatives to support and encourage mothers of moderate- and late-preterm babies in their lactation.

## Introduction

Worldwide, babies born moderate (MP)- to late (LP)-preterm (32^+0^ −36^+6^) account for >80% of all preterm babies born each year (1). It is becoming increasingly apparent that, compared with term babies, MP and LP babies are at higher risk of several complications following birth, including hypoglycaemia, jaundice, temperature instability, sepsis, and hospital readmission (2–9). They also are at an increased risk of worse long-term outcomes compared with term-born babies, including developmental delay (10), neurodevelopmental disability (11), need for special education (12) and behavioral problems (13). In babies born <32 weeks' gestation, better growth is associated with better neurodevelopmental outcomes (14), and growth is associated with nutrition (15). However, whether the same is true for MP and LP babies remains unknown. Given that the fetal brain continues to grow very rapidly during the final trimester, with brain volume at 32–34 weeks' gestation only 55–65% of the brain volume at term (16, 17), it is plausible that early nutrition and growth could also be important for optimizing neurodevelopmental outcomes of MP and LP babies.

One of the most significant issues facing clinicians following the birth of a MP or LP baby is how best to provide early nutritional support for optimal short- and long-term health. Breastmilk is the preferred nutrition for these babies but usually is not available in the first days after birth in sufficient quantities to meet the nutritional requirements, such as maintaining hydration and avoiding hypoglycaemia (18). Therefore, these requirements need to be met in other ways until sufficient breastmilk is available to meet the baby's physiological needs. The lack of robust evidence on which to base clinical practice is reflected in the great variation in the nutritional management of MP and LP babies (19–22). Common approaches include the provision of infant formula *via* a gastric feeding tube or provision of intravenous (IV) fluids (20) that may consist of dextrose alone or an amino acid solution with or without lipid emulsion. Provision of IV fluids means admission to a neonatal nursery, usually with separation of the mother–baby dyad, and the need for IV access. If IV fluids are commenced, how long one should wait for breastmilk supply to meet the baby's needs, thereby ensuring the baby only receives breastmilk rather than providing an alternative form of nutrition that may facilitate discharge and avoid the need for intravenous access, is a common dilemma (23).

The DIAMOND trial (DIfferent Approaches to MOderate & late preterm Nutrition: Determinants of feed tolerance, body composition, and development) is a randomized, controlled, factorial-design trial that aims to investigate the effects of different nutritional strategies in MP and LP babies on their body composition, time to full enteral feeds, and neurodevelopmental outcomes (24).

The objective of this study is to describe the actual nutritional management of a cohort of MP and LP babies who were randomized, as part of the DIAMOND trial, to nutritional management that stipulated MOM as the only enteral feed, with the provision of only IV fluids support until breastmilk supply met requirements. Clinicians were asked explicitly not to provide an alternative to MOM unless they believed that this was clinically indicated or the parents requested an alternative. We hypothesized that clinicians would consider that the smallest babies would benefit most from receiving only breastmilk, which is associated with a reduced risk of feed intolerance and necrotising enterocolitis (NEC) (25), although both are uncommon in MP and LP babies. We also hypothesized that parenteral nutrition (PN) would be considered by clinicians to provide more balanced nutrition than dextrose alone due to the administration of, at the least, additional protein. We therefore hypothesized that the smallest babies and those receiving PN would be more likely to transition successfully from IV fluids onto MOM only, and would be less likely to receive alternative forms of nutrition.

## Materials and Methods

In 2017, the DIAMOND trial began recruiting babies born between 32^+0^ and 35^+6^ weeks' gestation across 4 neonatal units in Auckland, New Zealand, all of which are accredited through the baby-friendly hospital initiative (26). Babies with IV line placed for clinical reasons (type of line access was according to local practice) and whose mothers intended to breastfeed were eligible. Consent was required within 24 h of birth and babies were randomized to three interventions in a factorial design, leading to eight possible conditions (24). The three interventions (factors to which babies were randomized) were: [factor 1] either dextrose or PN (at a minimum amino acid solution, with lipid emulsion at the medical team's discretion); [factor 2] MOM as the only enteral feed or, if breastmilk was insufficient to meet prescribed fluid requirements, a milk supplement (peer-to-peer donor expressed breastmilk or infant formula according to local practice) while waiting for MOM to reach desired volumes; and [factor 3] exposure to smell and taste of milk before tube feeds or not. The goal for all babies was to achieve full feeds with MOM. Pasteurized donor human milk was not available at the recruiting sites during the study period. Each of the recruiting hospitals had dedicated lactation consultants and the provision of lactation support and advice was as per local practice; no extra support was provided for mothers within the DIAMOND trial protocol, details of which can be found in the published protocol (24).

This study cohort comprises babies recruited between March 2017 and 2020 who, within factor 2, were randomized to receive MOM as the only enteral feed. The protocol specified that babies should remain on IV fluids until milk feeds of MOM met the prescribed fluid requirements without the need for further IV fluids. Babies could receive an alternative enteral nutrition if the clinical team felt they no longer could wait for sufficient MOM or at parental request. Babies were categorized according to whether they received MOM only or received alternative enteral nutrition to transition from IV fluids onto enteral feeds. Successful transition from IV fluids onto MOM only was defined as MOM being the sole enteral feeding at the time of first cessation of intravenous fluids.

Babies' birth characteristics and nutrition status up to discharge are summarized as median and range or as frequencies and percentages as appropriate. Simple Chi-square (or Fisher's exact test with small counts) and logistic regression were used to compare the differences in proportions between subgroups. Time to transition from IV fluids onto enteral feeds was compared between groups using analysis of variance. Time to receive alternative nutrition was evaluated using Kaplan–Meier survival curves and log-rank test for gestational age, birth characteristics, and IV fluid type commenced at birth. Statistical analysis was performed using SAS version 9.4 (SAS Institute Inc., Cary, NC, USA). All statistical tests were two-sided at a 5% level of significance.

The New Zealand Health and Disability Ethics Committee has given ethical approval for the DIAMOND study (16/NTA/90), and each participating site has institutional approval through local institutional review processes. The DIAMOND trial is endorsed by the IMPACT clinical trials network (https://impact.psanz.com.au) and prospectively registered (ACTRN12616001199404).

## Results

The cohort comprises 151 babies, 77 (51%) MP (32^+0^ −33^+6^ weeks' gestation) and 74 (49%) LP (34^+0^ −35^+6^ weeks' gestation) (Table 1). Of these, 65 (43%) received IV dextrose and 86 (53%) received PN, with 70% of the latter receiving IV lipid emulsion in addition to an amino acid solution. A total of 93 (63%) babies successfully transitioned from IV fluids onto MOM only. For the babies who received alternative enteral nutrition to transition from IV fluids, infant formula was given to all but 3; these babies received unpasteurised (peer-to-peer) donor-expressed breastmilk.

**Table 1:** Cohort characteristics.

**Characteristics**	**All (*n* = 151)**	**Moderate preterm (*n* = 77)**	**Late preterm (*n* = 74)**
**Gestational age at birth, (weeks)**	33.9 (32, 35.9)	32.9 (32, 33.9)	34.7 (34, 35.9)
**Sex–Boys**	90 (60)	46 (60)	44 (59)
**Birth weight (g)**	2,130 (1,250, 3,200)	1,910 (1,250, 2,720)	2,350 (1,315, 3,200)
**Small for gestational age**	18 (12)	5 (6)	13 (18)
**Birth by cesarean section**	95 (63)	48 (62)	47 (64)
**Hospital site**			
Auckland	62 (41)	33 (43)	29 (39)
Middlemore	49 (32)	28 (36)	21 (29)
North Shore	31 (21)	13 (17)	18 (24)
Waitakere	9 (6)	3 (4)	6 (8)
**Ethnicity**			
Caucasian	50 (33)	27 (35)	23 (31)
Māori	23 (15)	10 (13)	13 (17.5)
Asian	47 (31)	22 (29)	25 (34)
Pacific Island	31 (21)	18 (23)	13 (17.5)
**Multiples**	41 (27)	20 (26)	21 (28)
**Maternal diabetes**	23 (15)	13 (17)	10 (14)
**Maternal education**			
Lower secondary (< year 11)	15 (10)	9 (12)	6 (8)
Upper- (year 12 & 13) and Post-secondary Non-tertiary education	58 (38)	29 (38)	29 (39)
University degree	74 (49)	35 (45)	39 (53)
Other/Unknown	4 (3)	4 (5)	0 (0)
**Maternal age, (years)**	31 (15, 47)	32 (15, 47)	31 (20, 45)

*Data are presented as median (range) or N (%). Small for gestational age (SGA) is defined as birth weight below the 10th centile for gestational age on the Fenton growth charts (27)*.

Late preterm babies transitioned off IV fluids onto only enteral feeds more quickly than MP babies, but there was a significant interaction with sex, with LP boys taking the least time to transition off IV fluids and MP boys taking the most time (Table 2). Māori babies also transitioned off IV fluids more quickly than other ethnicities. There were no differences in time to transition off IV fluids between babies receiving different types of IV fluids, cared for in different hospitals, or with different modes of birth.

**Table 2:** Time to first cessation of IV fluids and provision of enteral feeds only.

	**All** **(*n* = 151)**	**MP** **(*n* = 77)**	**LP** **(*n* = 74)**
**Overall cohort** ^†^	4 (1, 11)	4 (1,11)	3 (1, 8)
**Sex**^Δ^, ^§^ Boys Girls	4 (1, 11) 4 (1, 10)	5 (1, 11) 4 (1, 10)	3 (1, 7) 3 (2, 8)
**Type of fluid** Dextrose Parenteral nutrition	4 (1, 9) 4 (1, 11)	4 (1, 9) 4 (1, 11)	3 (1, 8) 3 (1, 8)
**Small for gestational age^θ^** Yes No	5 (2, 8) 4 (1, 11)	5 (3, 8) 4 (1, 11)	4 (2, 8) 3 (1, 8)
**Birth by cesarean section** Yes No	4 (1, 8) 4 (1, 11)	4 (1, 8) 4 (1, 11)	3 (1, 8) 3 (1, 8)
**Hospital site**			
Auckland	4 (1, 10)	5 (1, 10)	3 (1, 8)
Middlemore	4 (1, 11)	4 (1, 11)	3 (1, 8)
North Shore	3 (1, 9)	4 (2, 9)	3 (1, 7)
Waitakere	3 (2, 10)	3 (2, 4)	8 (4, 10)
**Ethnicity** ^*^			
Caucasian	4 (1, 10)^a^	5 (1, 10)	3 (1, 7)
Māori	3 (1, 10)^b^	4 (1, 10)	3 (1, 4)
Asian	4 (1, 10)^a^	4 (3, 10)	3 (1, 8)
Pacific Island	4 (1, 11)^a^	4 (2, 11)	4 (1, 6)
**Multiples** Yes No	4 (1, 8) 4 (1, 11)	4 (3, 8) 4 (1, 11)	3 (1, 8) 3 (1, 7)
**Maternal diabetes** Yes No	4 (1, 8) 4 (1, 11)	4 (3, 8) 5 (1, 11)	3 (1, 5) 3 (1, 8)

*Data are presented as median (range) in days from birth. MP, moderate preterm; LP, late preterm.*

† *p <0.05 for MP vs. LP.*

Δ *p <0.05 for comparison between boys and girls in MP babies.*

§ *p <0.05 for interaction effect between sex and gestational age category.*

θ *p <0.05 for comparison between SGA and AGA in LP babies.*

* *p < 0.05 for effect of ethnicity. Data with different superscripts.*

*(^a, b^) are significantly different from each other (p < 0.05)*.

Multiples were less likely to achieve a successful transition to MOM only than singletons (46 vs. 67%, *p* = 0.02), and this was particularly the case for LP babies (33 vs. 68%, *p* = 0.007) (Table 3). Māori babies were less likely to achieve a successful transition to MOM only (43%) than Pacific (74%, *p* = 0.02) and Caucasian (70%, *p* = 0.03) babies. In LP babies only, the smallest (<1,500 g) and largest (>2,500 g) were more likely to achieve a successful transition to MOM only than babies with a birth weight between 1,500 and 2,500 g (*p* = 0.04). LP babies born by Cesarean section were less likely to achieve a successful transition to MOM only than LP babies born vaginally (49 vs. 74%, *p* = 0.03).

**Table 3:** Maternal and birth characteristics of babies who successfully transitioned onto only mothers own milk at the first cessation of IV fluids.

	**Proportion transitioning successfully to MOM**		
	**All (*n*=151) *n/N* (%)**	**MP (*n* = 77)** ***n/N* (%)**	**LP (*n* = 74) *n/N* (%)**
**Overall cohort**
	93/151 (62)	50/77 (65)	43/74 (58)
**Sex** Boys Girls	60/90 (67) 33/61 (54)	32/46 (70) 18/31 (58)	28/44 (64) 15/30 (50)
**Birthweight (g)**^Δ^ 1,000–1,499 1,500–1,999 2,000–2,500 >2,500	6/9 (67) 35/55 (64) 30/56 (54) 22/31 (71)	4/7 (57) 28/40 (70) 14/22 (63) 4/8 (50)	2/2 (100) 7/15 (47) 16/34 (47) 18/23 (78)
Ethnicity^*^ Caucasian Māori Asian Pacific	35/50 (70)^a^ 10/23 (43)^b^ 25/47 (53)^a, b^ 23/31 (74)^a^	20/27 (74) 4/10 (40) 13/22 (59) 13/18 (72)	15/23 (65) 6/13 (46) 12/25 (48) 10/13 (77)
**Hospital of birth** Auckland Middlemore North Shore Waitakere	42/62 (68) 30/49 (61) 16/31 (52) 5/9 (56)	23/33 (70) 16/28 (57) 9/13 (69) 2/3 (67)	19/29 (66) 14/21 (67) 7/18 (39) 3/6 (50)
**Cesarean section**^†^ Yes No	54/95 (57) 39/56 (70)	31/48 (65) 19/29 (66)	23/47 (49) 20/27 (74)
**Small for gestational age** Yes No	12/18 (67) 81/133 (61)	4/5 (80) 46/72 (64)	8/13 (62) 35/61 (57)
**Maternal diabetes** Yes No	12/23 (52) 81/128 (63)	7/13 (54) 43/64 (67)	5/10 (50) 38/64 (59)
**Maternal education** Lower secondary (< year 11) Upper (year 12 & 13) and Post-secondary Non-tertiary education University degree	7/15 (47) 36/58 (62) 50/74 (68)	4/9 (44) 21/29 (72) 25/35 (71)	3/6 (50) 15/29 (52) 25/39 (64)
**Multiples**^Δ^ Yes No	19/41 (46) 74/110 (67)	12/20 (60) 38/57 (67)	7/21 (33) 36/53 (68)

*Data are presented as n/N (%). MOM, mothers own milk; MP, moderate preterm; LP, late preterm.*

* *p < 0.05 for effect of variable across the whole cohort. Data with different superscripts (^a, b^) are significantly different from each other (p < 0.05).*

† *p < 0.05 for comparison between mode of birth groups in LP babies.*

Δ *p < 0.05 across the whole cohort*.

**Figure 1:**
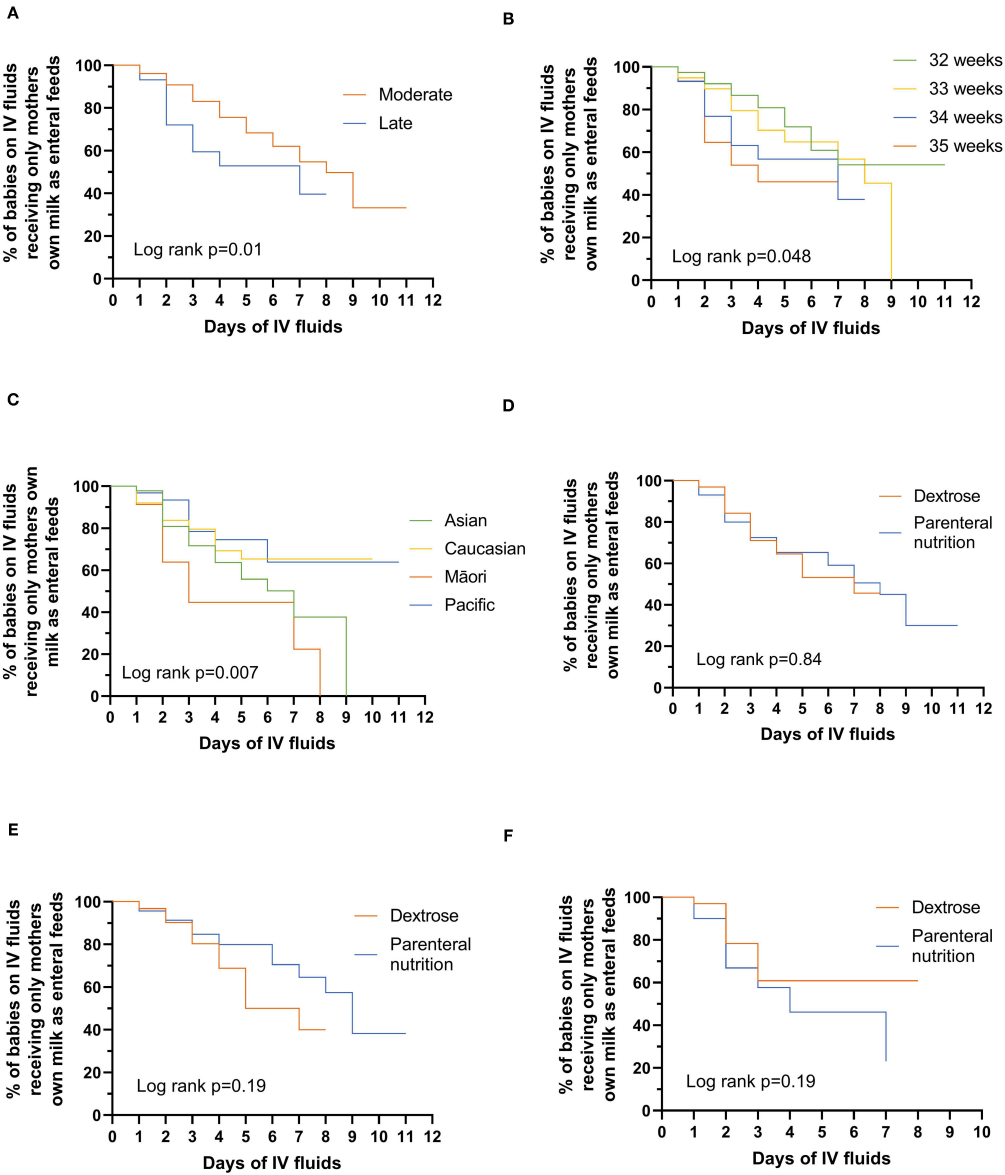
Days to alternative nutrition while on intravenous fluids by: **(A)** moderate vs. late preterm babies; **(B)** gestational age in weeks; **(C)** ethnicity; **(D)** whether babies are receiving dextrose or parenteral nutrition; **(E)** moderate-preterm babies receiving dextrose or parenteral nutrition; **(F)** late-preterm babies receiving dextrose or parenteral nutrition. Significant differences were tested using the log-rank test (two-sided at 5% level of significance).

In babies who did not successfully transition to MOM only, LP babies received alternative nutrition earlier than MP babies (Figure 1A, *p* = 0.01), with 47% of LP babies receiving alternative nutrition by day 4 compared with only 24% of MP babies. This effect was seen across the gestational age spectrum with 19, 30, 43, and 54% of babies born at 32, 33, 34, and 35 completed weeks', respectively, transitioning from IV to alternative nutrition by day 4 after birth (Figure 1B, *p* = 0.048). Māori babies received alternative nutrition earlier than babies of other ethnicities (Figure 1C, *p* = 0.007); by day 3, 55% of Māori, 22% of Pacific, 20% of Caucasian, and 28% of Asian babies were receiving alternative nutrition that was not MOM. Whether babies received PN or dextrose was not related to the time to receiving alternative nutrition (Figure 1D, *p* = 0.84), either overall or in MP and LP babies separately (Figures 1E,F).

Successful transition from IV fluids to MOM only was significantly associated with the type of feeding at discharge from the hospital. Of those babies discharged receiving exclusively breastmilk, 60% were exclusively breastfed, 38% breast and bottle-fed, and 2% were exclusively bottle-fed. Of babies who successfully transitioned to MOM only, 85% (*n/N* = 79/93) were discharged receiving exclusively breastmilk compared with 53% (*n/N* = 31/58) of those who did not [OR (95% confidence intervals) 4.9 (2.3–10.6), *p* < 0.0001]. Similarly, 86% (*n/N* = 73/85) of babies who received only MOM in the first week after birth were discharged from hospital receiving exclusively breastmilk compared with only 56% (n/N = 37/66) of those who received alternative nutrition within the first week [OR = 4.8 (2.2–10.4), *p* < 0.0001]. The type of feeding at discharge was significantly different based on ethnicity, where 84% of Caucasian (*n/N* = 42/50), 75% of Pacific (*n/N* = 23/31), 70% of the Asian (*n/N* = 33/47), and 52% of Māori (*n/N* = 12/23) babies were receiving exclusively breastmilk at discharge (*p* = 0.04). Māori babies were significantly less likely to receive exclusively breastmilk at discharge compared with Caucasian babies [OR = 0.2 (0.1–0.6), *p* < 0.01].

## Discussion

The World Health Organization recommends exclusive breastfeeding for all babies, and this is even more important for moderate- to late-preterm babies who are at an increased risk of adverse health outcomes, both in the short- and long-term. The focus of this cohort study was the factors that were associated with the choice of nutrition received at the first cessation of IV fluids. Our findings also confirmed that these choices about early nutrition are associated with feeding at discharge and therefore are likely to be reflected in feeding type at home.

The nutritional plan for all babies in this cohort was for them to receive only MOM: mothers had stated that they intended to breastfeed; babies already had intravenous access secured, and they were randomized to a condition that stipulated nutritional support with IV fluids until MOM supply met baby's needs or unless the attending clinician or parents no longer felt able to do so. Despite this, almost 40% of babies received a breastmilk alternative, almost exclusively infant formula. A limitation is that the trial protocol did not collect the reason for the introduction of an alternative form of nutrition or whether this was a medical or parental decision.

We hypothesized that the smallest preterm babies would be more likely to transition onto MOM only to avoid exposing these babies to infant formula. However, this was not the case, as babies of lower birth weight or who were small-for-gestational age (SGA) were not less likely to receive formula. This may indicate that clinicians are comfortable using infant formula in smaller babies given that the risk of necrotising enterocolitis is low in MP compared with extremely preterm babies (28). The high level of formula use is consistent with our survey of nutritional management in MP and LP babies (22). The provision of infant formula to transition off IV fluids was much more likely in multiples than singletons, consistent with the published literature (29).

We also hypothesized that babies randomized to PN would be more likely than babies randomized to IV dextrose to transition successfully to MOM only, as dextrose provides only carbohydrates, leading inevitably to an accumulating nitrogen deficit (30). It has been proposed that this nitrogen deficit followed by restoration of adequate nutrition may contribute to altered body composition of MP and LP babies (31), resulting in a greater fat accumulation, inadequate accretion of lean body mass, and a higher fat mass percent at term-equivalent age (31, 32). However, PN provides protein (and fat when lipid emulsion also is provided, as was the case for 70% of babies receiving PN) in addition to carbohydrate, and we expected that this might encourage clinicians to delay the introduction of other protein sources. However, our findings did not support this hypothesis.

Although there was no statistical difference in the proportion of MP and LP babies transitioning successfully onto MOM, MP babies who received alternative forms of nutrition did so later than LP babies. This may reflect a number of factors including a greater willingness to commence infant formula in the more mature babies, a desire for less medicalisation in well LP babies, the pressure to limit the separation of the mother–infant dyad, pressure on bed status, and perhaps the acknowledgment that, as MP babies are likely to require at least 1–2 weeks in the newborn nursery, there is more time to wait for successful transition onto MOM. However, it is unclear why such factors did not lead to an overall difference in the proportion of MP and LP transitioning successfully to MOM.

Lactogenesis II, the secretory activation phase, typically occurs 48–72 h after birth and, following a normal term birth, breastmilk begins to be produced in significant quantities from day 4 post-birth (33). Delayed lactogenesis, defined as occurring after 72 h (34), is well described with stress and anxiety (35, 36), Caesarean section (37), diabetes and obesity (38), and following preterm birth (39). Therefore, if the goal is for babies to receive only MOM and avoid formula whenever possible, clinicians must be willing to wait for >4 days before commencing an alternative form of nutrition; this occurred in ~70% of MP but only 50% of LP babies.

The lack of donor breastmilk use in this study reflects the lack of a donor breastmilk bank accessible by the recruiting sites. However, donor unpasteurised breastmilk is sometimes provided through a mother screening and sharing system within the individual units and is most often used in the more preterm infants at a higher risk of NEC (40). With the high use of infant formula and clinician preference for enteral fluids rather than IV fluids, this does highlight the need for robust evidence on whether donor breastmilk in MP and LP babies has health benefits and is cost-effective.

Receiving milk other than MOM and, indeed, doing so only in the first week after birth, was negatively associated with breastmilk feeding at discharge. Rates of breastfeeding, the ability to exclusively breastfeed, and breastmilk production are well documented to be challenging to mothers of MP and LP babies (41–43). However, supportive care programs to encourage and support breastfeeding have been shown to improve breastmilk provision during admission, decrease the use of formula, and increase the rates of exclusive breastfeeding in moderate to late preterm infants (44–46). Therefore, there may be an opportunity for quality initiatives to increase breastmilk provision, particularly in the first week after birth in this high-risk group.

Perhaps the most striking finding of this study was that Māori babies, the indigenous peoples of New Zealand, transitioned onto formula significantly earlier than babies of other ethnicities. Māori babies also were least likely to achieve breastmilk feeding on discharge, a concerning finding given the risk of poor health outcomes associated with low breastfeeding rates and the protective effects of breastfeeding on sudden infant death syndrome (47), childhood obesity (48), and respiratory infections (49), all of which are over-represented in Māori children. We are unable to determine from this study the reasons underlying these findings, which require further investigation. Possibilities include social factors impacting the mother's ability to visit and express regularly, less access to resources and equipment that support expressing breastmilk, and the knowledge that it might be difficult to continue completely breastfeeding after discharge. However, there may also be larger issues involved including a healthcare system that may not be aligned with the Māori perspective on healthcare, and an unconscious bias or systemic racism within the healthcare system.

## Conclusion

Even when the intention is to provide only MOM within the context of a randomized control trial, this goal is not achieved for many MP and LP babies. Formula is often provided even before lactogenesis II is likely to be established, suggesting that mothers are not given the opportunity to establish a milk supply before breastmilk alternatives are provided. Māori babies were significantly more likely to receive formula, to be given formula at an earlier age, and were least likely to be breastfed at discharge. Further research should address the reasons behind these findings and find measures to address them.

## Data Availability Statement

The raw data supporting the conclusions of this article will be made available by the authors, without undue reservation.

## Ethics Statement

The studies involving human participants were reviewed and approved by the New Zealand Health and Disability Ethics Committee (16/NTA/90). Written informed consent to participate in this study was provided by the participants' legal guardian/next of kin.

## Author Contributions

TA conceptualized and designed the study and protocol, drafted the initial manuscript, obtained funding for the study, contributed to the acquisition and interpretation of the data, reviewed, and revised the manuscript. FB conceptualized and designed the study and protocol, obtained funding for the study, contributed to the interpretation of the data, reviewed and revised the manuscript. YJ contributed to the study design, protocol development, and analysis of the data. MM, JH, JA, and CW contributed to protocol development and have commented on all drafts of manuscript. MM was involved in the acquisition of the data and commented on all drafts of manuscript. All authors have approved the final manuscript as submitted and agree to be accountable for all aspects of the work.

## Funding

The DIAMOND trial is funded by the Health Research Council of New Zealand (16/605) and Counties Manukau Health (number 269).

## Conflict of Interest

The authors declare that the research was conducted in the absence of any commercial or financial relationships that could be construed as a potential conflict of interest.

## Publisher's Note

All claims expressed in this article are solely those of the authors and do not necessarily represent those of their affiliated organizations, or those of the publisher, the editors and the reviewers. Any product that may be evaluated in this article, or claim that may be made by its manufacturer, is not guaranteed or endorsed by the publisher.
